# Variation of rice starch structure and physicochemical properties in response to high natural temperature during the reproductive stage

**DOI:** 10.3389/fpls.2023.1136347

**Published:** 2023-02-14

**Authors:** Debao Tu, Yang Jiang, Akram Salah, Min Xi, Mingli Cai, Bo Cheng, Xiaosong Sun, Cougui Cao, Wenge Wu

**Affiliations:** ^1^ Rice Research Institute, Anhui Academy of Agricultural Sciences, Hefei, China; ^2^ National Key Laboratory of Crop Genetic Improvement, MOA Key Laboratory of Crop Physiology, Ecology and Cultivation (The Middle Reaches of Yangtze River), Huazhong Agricultural University, Wuhan, China; ^3^ Key Laboratory of Plant Functional Genomics of the Ministry of Education, Co-Innovation Center for Modern Production Technology of Grain Crops, College of Agriculture, Yangzhou University, Yangzhou, China

**Keywords:** chain length distribution, pasting properties, crystallinity, taste value, chalkiness degree

## Abstract

Climate warming affects rice growth at different phenological stages, thereby increasing rice chalkiness and protein content and reducing eating and cooking quality (ECQ). The structural and physicochemical properties of rice starch played important roles in determining rice quality. However, differences in their response to high temperature during the reproductive stage have been rarely studied. In the present study, they were evaluated and compared between two contrasting natural temperature field conditions, namely, high seasonal temperature (HST) and low seasonal temperature (LST), during the reproductive stage of rice in 2017 and 2018. Compared with LST, HST significantly deteriorated rice quality, including increased grain chalkiness, setback, consistence, and pasting temperature and reduced taste values. HST considerably reduced the total starch and increased the protein content. Likewise, HST significantly reduced the short amylopectin chains [degree of polymerization (DP) <12] and increased the long amylopectin chains (DP > 12) and relative crystallinity. The starch structure, total starch content, and protein content explained 91.4%, 90.4%, and 89.2% of the total variations in pasting properties, taste value, and grain chalkiness degree, respectively. In conclusion, we suggested that rice quality variations were closely associated with the changes in chemical composition content (total starch and protein content) and starch structure in response to HST. These results indicated that we should improve the resistance of rice to high temperature during the reproductive stage to improve the fine structure of rice starch in further breeding and practice.

## 1 Introduction

Rice is the most important food resource to meet the caloric and nutritional requirements of humans (Hu et al., 2017; Lin et al., 2020). Therefore, improving grain yield potential is the major goal of almost all rice breeders to increase rice production to meet market demands (Zhang et al., 2016a). Rice quality is a complex trait that reflects the aroma, taste, milling, appearance, eating and cooking quality (ECQ), and nutritional quality, which are closely associated with the structure and properties of rice starch such as amylose content, amylopectin chain-length distribution, crystallinity, and gelatinization temperature (Cai et al., 2015; Zhang et al., 2016a; Tao et al., 2018). In addition to genetic effects, rice quality is also significantly affected by different environmental conditions (Yao et al., 2019). For example, rice quality can vary inexplicably from different years and even from different sowing dates (Huang et al., 2013; Bai et al., 2019).

Variations in growth temperature might be an important reason for differences in rice quality (Krishnan et al., 2011; Huang et al., 2013). Numerous studies have shown that high temperature significantly reduced milled rice rate, increased chalky grain rate and chalkiness degree, and altered the physicochemical properties of rice starch, i.e., reduced amylose content, increased pasting properties, and elevated gelatinization temperature (Lin et al., 2014; Nisar et al., 2015; Zhang et al., 2016b). Furthermore, they also found that high temperatures were associated with a change in starch structure in the ways described, such as reduced long-chain amylose and short-chain amylopectin and increased long-chain amylopectin and crystallinity, which were significantly associated with the changes in rice quality (Zhang et al., 2016a; Yao et al., 2019). However, most of these studies focused on the influence of high temperature during flowering and grain filling stages.

In addition to the flowering and grain filling stages, the reproductive stage (from panicle initiation to the heading stage) which is involved in pre-anthesis assimilate accumulation at early panicle development also plays a key role in determining the rice starch physicochemical properties (Lin et al., 2020). It has been reported that extremely high-temperature conditions often occurred from early July to mid-August, which would affect the rice grain development and quality in the Yangtze River Valley (Tao et al., 2012; Tao and Zhang, 2013; Deng et al., 2015; Cheng et al., 2020). Thus, it is necessary to clearly understand the influence of high natural temperature during the reproductive stage on rice quality. Previous studies reported that high temperature during the reproductive stages resulted in a high chalky grain rate, high chalkiness degree, and high protein content (Cheabu et al., 2018; Zhen et al., 2019; Lin et al., 2020). Nevertheless, the differences in rice starch structure and the physicochemical properties’ response to high temperatures during the reproductive stage have been rarely studied.

Furthermore, previous studies were usually conducted under controlled temperature chambers. This approach is limited in that it does not necessarily reproduce field conditions (Huang et al., 2013). In the present study, rice quality, structure, and physicochemical properties were compared between two contrasting natural temperature conditions during the reproductive stage. The main objectives of this study were to 1) evaluate the effect of high natural temperature during the reproductive stage on the structure and physicochemical properties of rice starch and 2) explore the relationship among rice quality, starch structure, and physicochemical properties.

## 2 Materials and methods

### 2.1 Plant materials, site description, and growth conditions

**Figure 1:**
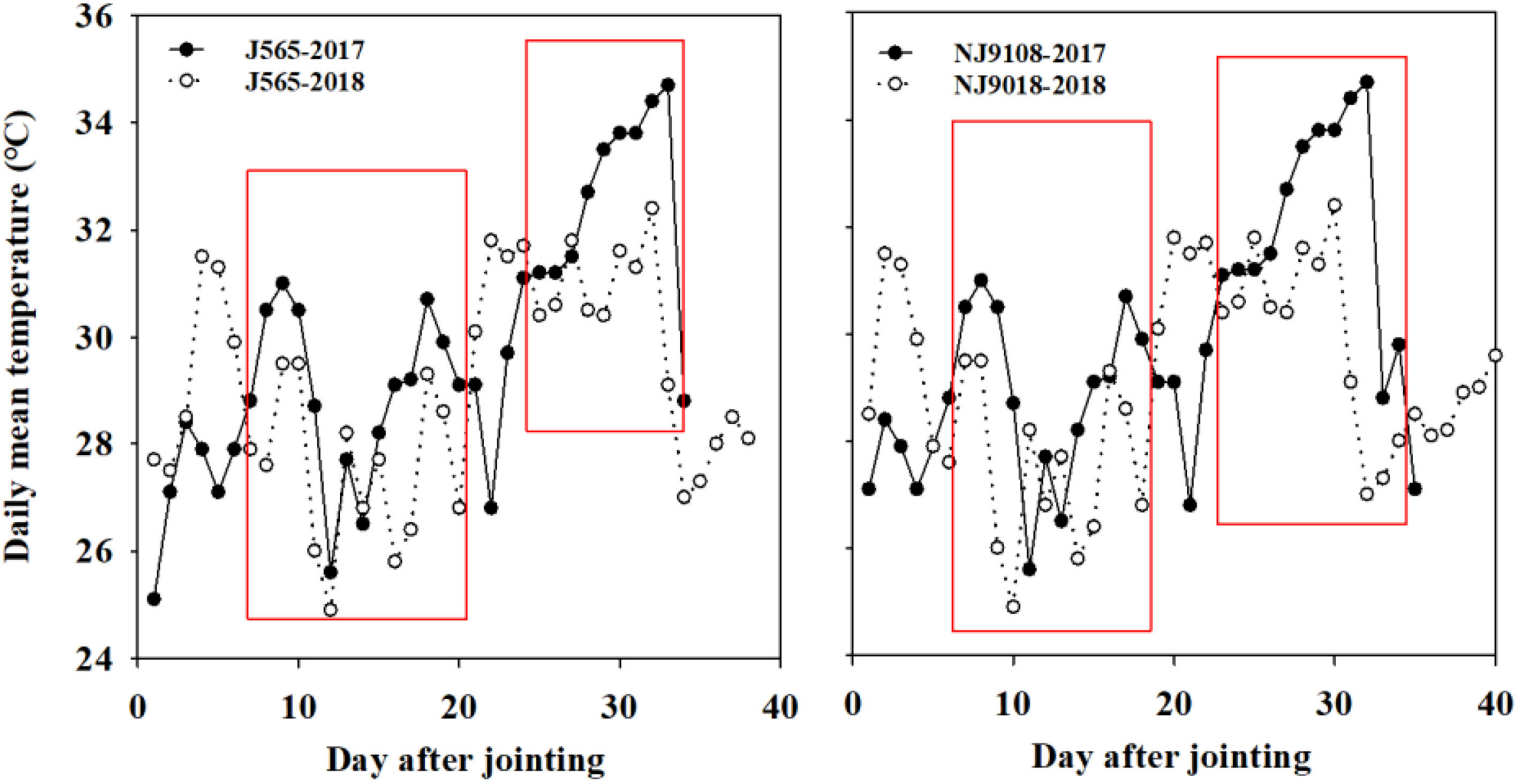
Daily mean temperature conditions during the reproductive stage in 2017 and 2018. J565 and NJ9108 represent cultivars Jing-565 and Nanjing-9108. The red boxes indicated the significant difference of mean daily temperature between the two contrasting growth seasons.

In 2017 and 2018, field experiments were carried out at the farm of Xiaowan Village, Zaoyang, Hubei Province (32.0°N, 112.8°E). The soil was alluvial sandy loam with 34.3 g kg^−1^ of organic matter content, 1.58 g kg^−1^ of total nitrogen, 8.75 mg kg^−1^ of Olsen-P, and 56.6 mg kg^−1^ of exchangeable K. The experiments were arranged in a randomized complete block design with three replications. Two japonica rice varieties with excellent sensory properties, i.e., Nanjing-9108 (NJ9108) and Jing-565 (J565), were used in the study. These cultivars were susceptible to high temperatures. The field management practices were similar in 2017 and 2018. The temperature conditions are shown in **Figure S1**. The daily temperature during the reproductive stage in 2017 was higher than in 2018, while temperatures during the other stages (vegetative and grain filling stages) were similar (**Table 1**). The average daily temperature during the productive stage in 2017 was 0.6-0.7°C higher than that in 2018. Moreover, the daily mean temperature in 2017 was 0.5-5.6°C higher than that in 2018, which almost continued for more than 20 days (**Figure 1**). Our data were divided into four samples with three replications, namely, J565-HST, J565-LST, NJ9108-HST, and NJ9108-LST. HST and LST mean high seasonal temperature and low seasonal temperature, respectively, during the reproductive stage in 2017 and 2018.

**Table 1:** Growth duration and mean temperature during different phenological stages in different situations.

Treatment	Sowing date (year/m/d)	Jointing date (year/m/d)	Heading date (year/m/d)	Maturity (year/m/d)	VT (°C)	RT (°C)	GT (°C)	GT20 (°C)
J565-HST	2017/4/15	2017/6/26	2017/7/28	2017/9/28	23.8	29.7	25.9	29.0
J565-LST	2018/4/19	2018/6/23	2018/7/31	2018/10/4	23.8	29.1	25.8	29.1
NJ9108-HST	2017/4/15	2017/6/27	2017/7/30	2017/9/29	23.8	29.9	25.7	29.0
NJ9108-LST	2018/4/19	2018/6/25	2018/8/4	2018/10/6	23.9	29.2	25.5	29.0

VT, RT, GT, and GT20 represent the average daily temperature during the vegetative stage, reproductive stage, grain filling stage, and the first 20 days of grain filling stage, respectively. HST and LST represent high seasonal temperature and low seasonal temperature, during the reproductive stage in 2017 and 2018. J565 and NJ9108 represent cultivars Jing-565 and Nanjing-9108.

### 2.2 Measurement of milling and appearance quality

In the center of the plot, 5-m^2^ plants were harvested at the maturity stage. After that, the rice was threshed and air-dried. Seeds were stored for at least 3 months in a room temperature condition, and then they were hulled and milled by using a rice hulling machine (BLH-3250) and a rice polishing machine (Pearlest, Kett, Japan). Finally, the appearance quality (chalkiness degree) of the milled rice was measured by using a grain appearance analysis system (Epson Expression 1680 Professional, Epson, Japan) and an image analysis software.

### 2.3 Determination of total starch, apparent amylose, and protein contents

The total starch content was determined by the optical rotation method of Lu (2011). First, the 2.5-g rice flour sample was added to 40 ml of 85% ethanol, the suspension was centrifuged at 3,000 r/min for 10 min, and then the supernatant was removed. The above operation was repeated four times. Afterward, the residue was transferred into a 100-ml conical flask using 50 ml of 1.128% HCl and heated in a boiling water bath for 15 min. After cooling, the solution was added to 1 ml of 30% ZnSO_4_ and 1 ml of 15% potassium ferrocyanide and then was filtrated to obtain a clear liquid. The filtrate was put into a polarimeter (WZZ-2S, China) and the total optical rotation was measured. The starch content was calculated by its total optical rotation according to the method of Lu (2011).

The apparent amylose content (AAC) was determined by the iodine blue colorimetric method (Sandhu and Singh, 2007). The 100-mg rice flour was transferred to a 100-ml conical flask. Then, 1.0 ml of 95% ethanol and 9.0 ml of 1.0 mol/L NaOH were added. The suspension was mixed and heated in a boiling water bath for 10 min and cooled to room temperature. The solution was transferred to a 100-ml volumetric flask and distilled water was added to a constant volume (100 ml). Then, 0.5 ml of the solution sample was transferred into a covered colorimetric tube with 5 ml water and 0.1 ml of 1 mol/L acetic acid and then 0.2 ml of 0.2% iodine solution was added. Finally, its absorbance at 620 nm was measured with a spectrophotometer (TECAN Infinite M200, Switzerland). The AAC was determined from a standard curve established using a mixed solution of amylose and amylopectin.

The 0.2-g rice flour was transferred into a digestion cube, and 5 ml of concentrated H_2_SO_4_ was added overnight. The suspension was added with three to four drops of H_2_O_2_ and then heated at 320°C for 10-15 min by using a digestion oven (Foss Tecator, Hillerod, Denmark). After cooling, two to three drops of H_2_O_2_ were added into the suspension and heated at 200°C for 5 min again. This step was repeated two to three times until the liquid is transparent. The protein content was determined according to the Kjeldahl method, and a nitrogen conversion factor of 5.95 was used to calculate the crude protein content of the milled rice (Zhang et al., 2016a).

### 2.4 Measurement of taste value and pasting properties

An RCTA-11A Taste Analyzer (Satake, Japan) was used to determine the taste value. The 30-g milled rice sample was washed with water for 20 s. Then, it was transferred into a pot and added with water, following the ratio of rice to water of 1:1.35. It was soaked for 30 min and steamed for 40 min. After that, it was cooled at room temperature for 1.5 h to examine the taste value, including appearance, hardness, stickiness, and balance degree of the cooked rice. High taste value often indicates better taste quality (Shi et al., 2022). The viscosity profiles of starch (peak viscosity, trough viscosity, final viscosity, and pasting temperature) were determined by a rapid viscosity analyzer (RVA) (Newport Scientific Instruments Co., Ltd., Australia). Thermal Cycle for Windows (TCW) was used for the analysis according to the methods of (Zhang et al., 2016a). A 3.0-g rice flour sample was dispersed with 25 ml of water. It was heated at 50°C for 1 min and heated to 95°C within 3.8 min. After that, it was heated at 95°C for 2.5 min and cooled to 50°C within 3.8 min. Finally, it was stirred (160 r/min) at 50°C for 1.4 min.

### 2.5 Starch isolation

The rice starch sample was isolated following the method of Zhu et al. (2016). The 20-g rice flour sample was transferred into a conical flask and added with 50 ml of a 0.45% sodium metabisulfite aqueous solution and 50 mg/g of alkaline protease. The homogenate was placed at room temperature condition for 24 h. After that, it was sieved with a 200-mesh sieve and the residue was rinsed with deionized water. The filtered starch slurry was transferred into a centrifuge tube and then centrifuged for 10 min (3,000 r/min), and then the supernatant and the faintly colored upper residual layer were removed. The remnant was mixed with 20 ml of deionized water and centrifuged again. This step was repeated approximately five times. Finally, the remnant was dried at 30°C and sieved (200 mesh).

### 2.6 Determination of the fine structure of starch

First, the purified starch was debranched by using isoamylase (EC 3.2.1.68 Sigma). After that, high-performance anion-exchange chromatography (HPAEC) and amperometric detection were used to determine the length distributions of the starch branch chains similar to the method proposed by (Zhang et al., 2016a); 150 mM of NaOH (eluent A) and 150 mM of NaOH with 500 mM of sodium acetate were prepared. The gradient procedure was as follows: 35% of eluent B at 0 and 2 min, 60% at 17 min, and 80% at 40 min. The separations were conducted under 25°C conditions with a flow rate of 0.5 ml/min. Finally, the peak for each degree of polymerization (DP) was attained.

### 2.7 Measurement of X-ray powder diffraction

The X-ray diffractometer (D8 Advance, Bruker-AXS, Germany) was used to analyze X-ray powder diffraction (XRD). The sample was canned over the 2*θ* range from 5° to 40° (1° min−^1^ rate and 0.02° step size). Relative crystallinity (%) was determined as the ratio of total crystalline peak areas to that of the total diffractogram by using the Origin 2018 software.

### 2.8 Statistical analysis

The data in all of the tables and figures represented the average values of triplicate experiments. The SPSS 25.0 Statistical Software Program performed ANOVA, Tukey’s test (*P* < 0.05), and Pearson correlation analysis. The principal component analysis was performed using Canoco 5. The figures were displayed by SigmaPlot 10.0 (Systat Software Inc., San Jose, CA, USA).

## 3 Results

### 3.1 Variations of grain chalkiness degree, taste value, and RVA profiles under high natural temperature during the reproductive stage

**Figure 2:**
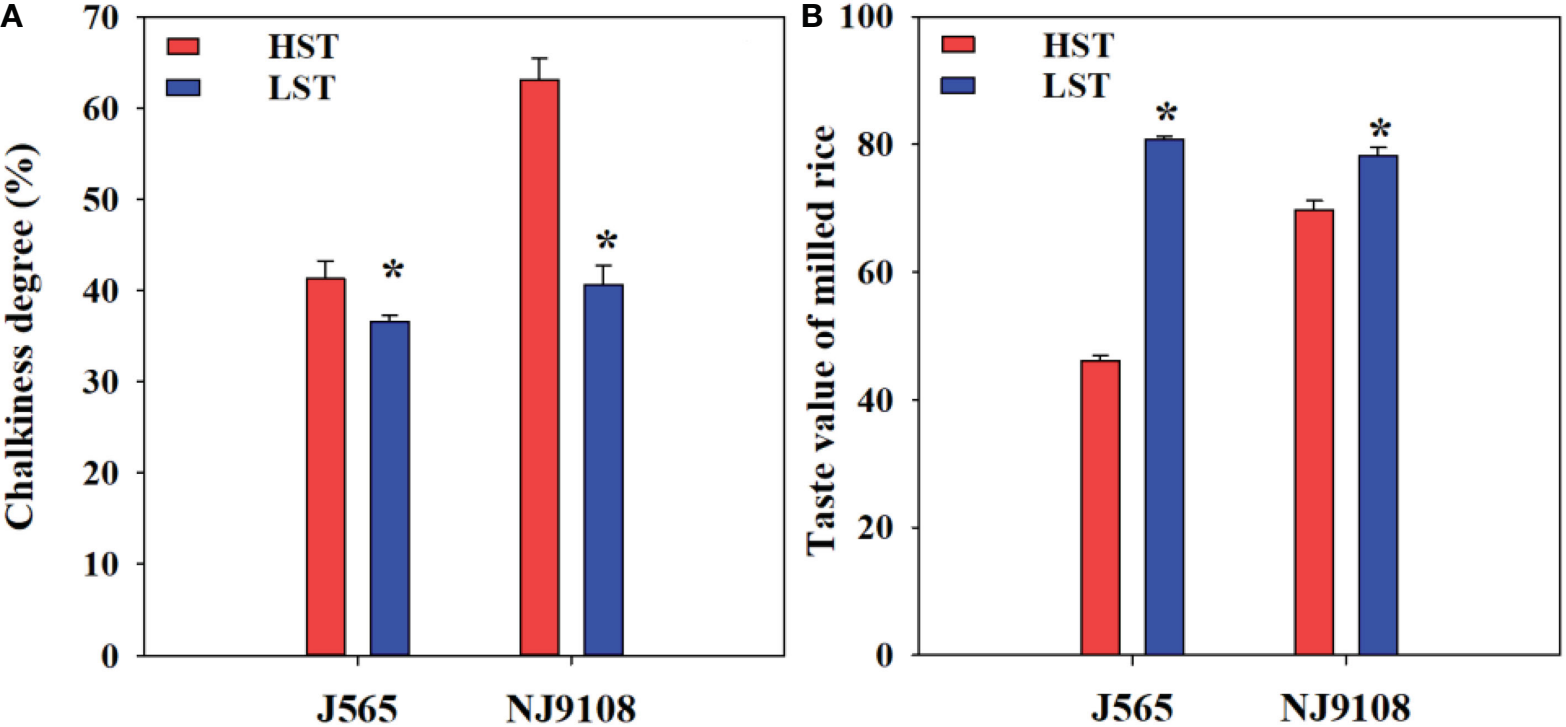
Chalkiness **(A)** and taste value of milled rice **(B)** in the two growth seasons. HST and LST represent high seasonal temperature and low seasonal temperature, during the reproductive stage in 2017 and 2018. J565 and NJ9108 represent cultivars Jing-565 and Nanjing-9108. * represents significant difference between HST and LST at the level of *P* < 0.05.

**Figure 3:**
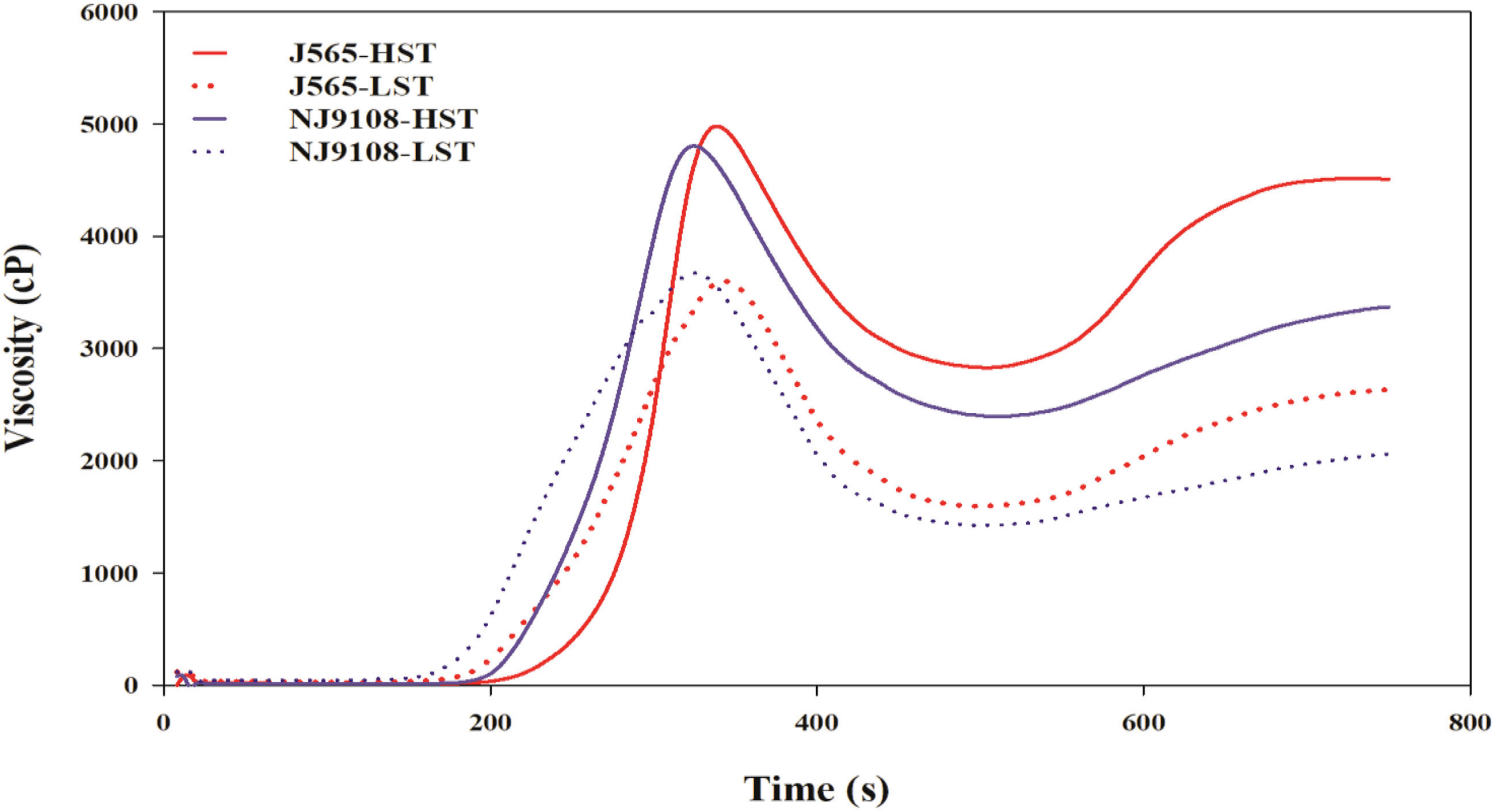
The RVA profiles in the two growth seasons. HST and LST represent high seasonal temperature and low seasonal temperature, during the reproductive stage in 2017 and 2018. J565 and NJ9108 represent cultivars Jing-565 and Nanjing-9108.

Chalkiness degree and taste value demonstrated significant differences for both cultivars between the two growth seasons (**Figure 2**). Compared with LST, HST markedly decreased the taste value and increased the chalkiness degree. To predict the texture of milled rice, the RVA was measured in the present study. As shown in **Figure 3**, there were great differences between the two growth seasons. The rice flour in HST had greater viscosity than that in LST. Furthermore, the pasting temperature, setback, and consistence were significantly higher in HST than those in LST (**Table 2**). These results indicated that high natural temperature during the reproductive stage significantly affects rice quality, including the increased chalkiness degree and reduced ECQ.

**Table 2:** Difference of rice flour pasting properties in the two growth seasons.

Treatment	Breakdown	Setback	Consistence	Pasting temperature
J565-HST	2,116.0 ± 35.2 a	−388.0 ± 88.6 a	1,728.0 ± 53.5 a	81.2 ± 0.5 a
J565-LST	1,759.0 ± 125.5 b	−615.5 ± 53.0 b	1,025.7 ± 37.6 b	75.9 ± 1.4 b
NJ9108-HST	2,168.3 ± 153.5 a	−1,142.0 ± 98.7 c	1,026.3 ± 45.8 b	77.8 ± 0.9 b
NJ9108-LST	2,231.3 ± 107. 2 a	−1,570.0 ± 122.5 d	661.3 ± 26.3 c	71.3 ± 0.9 c

HST and LST represent high seasonal temperature and low seasonal temperature, during the reproductive stage in 2017 and 2018. J565 and NJ9108 represent cultivars Jing-565 and Nanjing-9108. Values in the same column with different letters are significantly different (P < 0.05).

### 3.2 Effects of high natural temperature during the reproductive stage on total starch, apparent amylose, and protein contents

**Figure 4:**
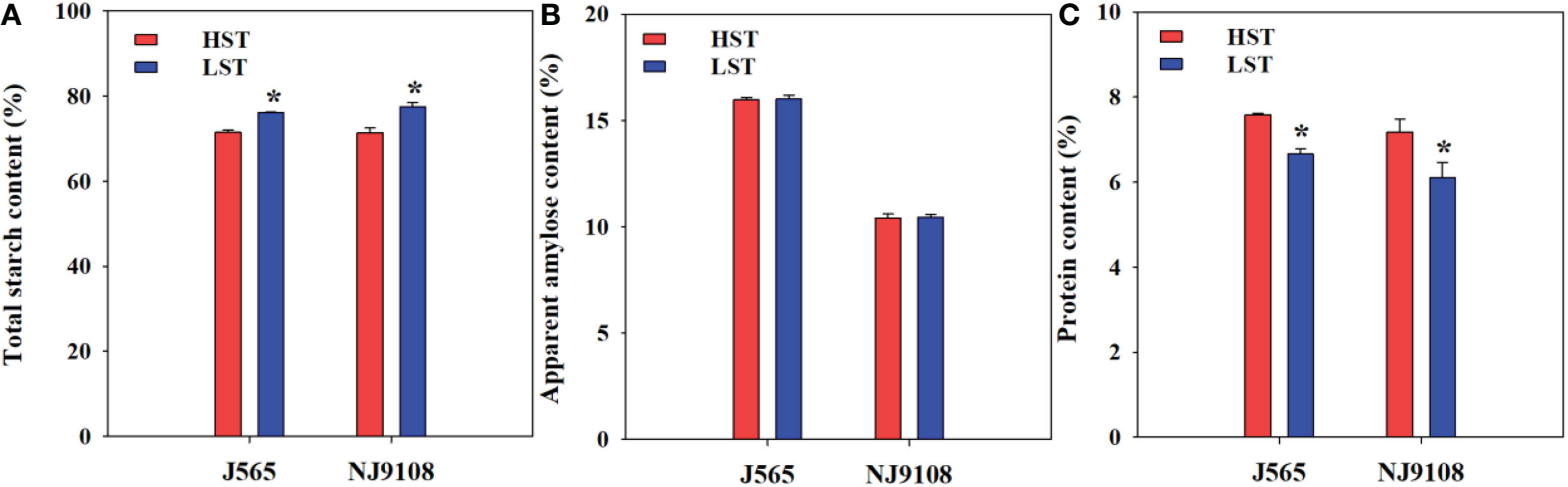
Total starch content **(A)**, apparent amylose content **(B)**, and protein content **(C)** in the two growth seasons. HST and LST represent high seasonal temperature and low seasonal temperature, during the reproductive stage in 2017 and 2018. J565 and NJ9108 represent cultivars Jing-565 and Nanjing-9108. * represents significant difference between HST and LST at the level of *P* < 0.05.

The major components were also investigated and compared between the two growth seasons in the present study (**Figure 4**). The AAC showed a slight difference between HST and LST in the present study. Regardless of the AAC, HST significantly decreased total starch but increased protein content. These results suggested that high natural temperature during the reproductive stage markedly reduced total starch and enhanced the protein content.

### 3.3 Influences of high natural temperature during the reproductive stage on the chain length distribution of debranched amylopectin

**Figure 5:**
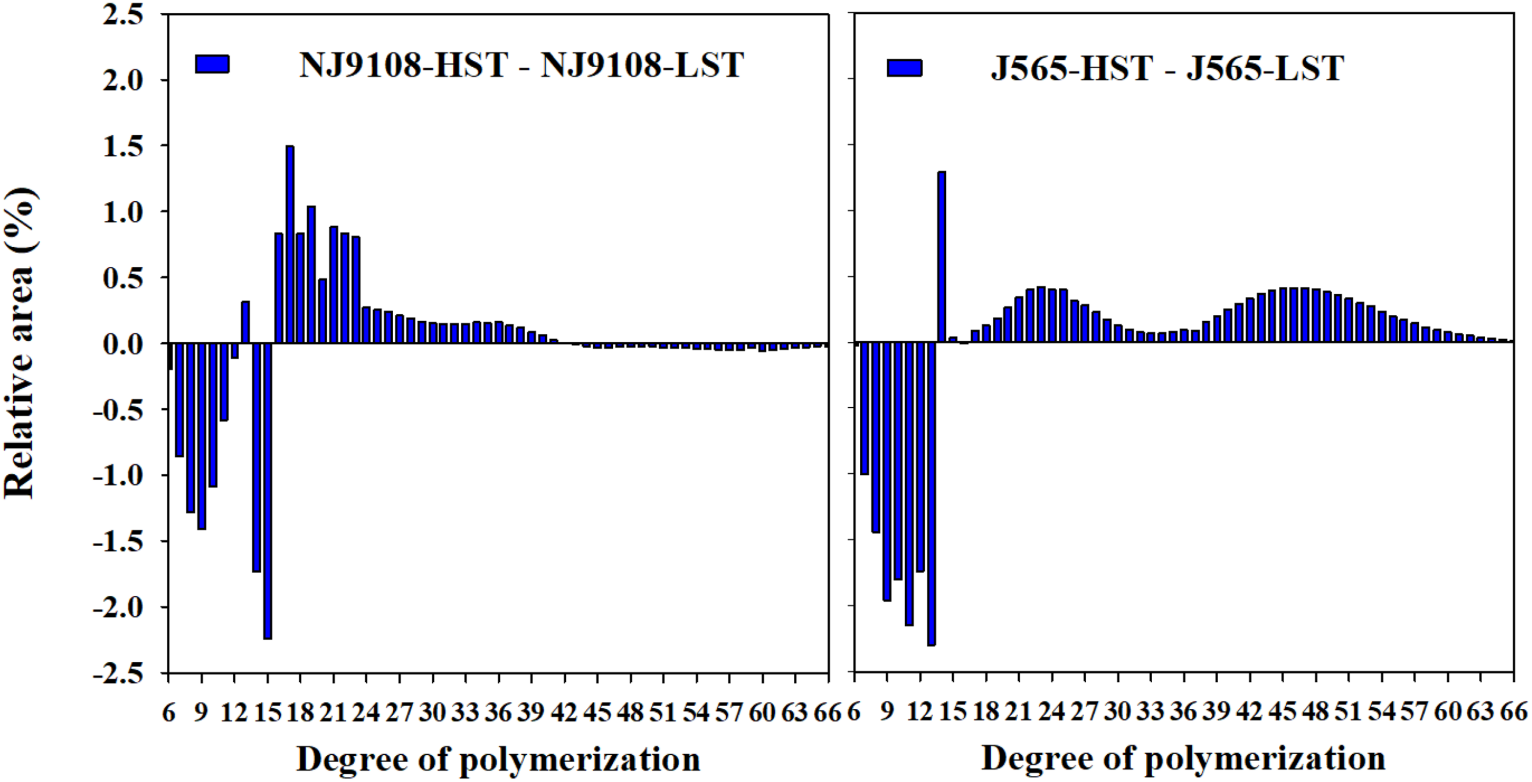
Differences in amylopectin chain length distribution between the two growth seasons. HST and LST represent high seasonal temperature and low seasonal temperature, during the reproductive stage in 2017 and 2018. J565 and NJ9108 represent cultivars Jing-565 and Nanjing-9108.

In the present study, the fractions of debranched amylopectin were analyzed by HPACE. As demonstrated in **Figure 5**, differences in the degree of DP of the two cultivars between two HST and LST presented similar tendencies. Branch chains could be classified into four chain types, namely, A chains (DP 6-12), B1 chains (DP 13-24), B2 chains (DP 25-36), and B3+ chains (DP ≥ 37). HST significantly decreased the A chains but noticeably increased the B1 and/or B2 chains (**Table 3**). The high natural temperature during the reproductive stage significantly decreased the rate of short chains (DP ≤ 12) to long chains (DP > 12) (S/L) and increased the proportion of long amylopectin chains.

**Table 3:** Branch-chain length distributions of debranched amylopectin of starch in the two growth seasons.

Variety	DP (6-12)	DP (13-24)	DP (25-36)	DP (≥37)	S/L
J565-HST	20.70 ± 1.09 c	52.37 ± 1.41 a	13.52 ± 1.44 b	13.40 ± 1.02 a	0.26 ± 0.02 c
J565-LST	30.82 ± 0.60 a	51.10 ± 0.60 a	11.44 ± 0.14 c	6.64 ± 0.14 b	0.44 ± 0.01 a
NJ9108-HST	19.85 ± 1.05 c	51.46 ± 1.21 a	15.17 ± 0.49 a	13.49 ± 1.78 a	0.25 ± 0.01 c
NJ9108-LST	25.37 ± 0.85 b	47.66 ± 0.50 b	13.02 ± 0.60 b	13.94 ± 0.71 a	0.34 ± 0.01 b

HST and LST represent high seasonal temperature and low seasonal temperature, during the reproductive stage in 2017 and 2018. S/L means the rate of short chains (DP < 12) to long chains (DP > 12). Data are expressed as the mean ± SD (n = 3). Values in the same column with different letters are significantly different (P < 0.05).

DP, degree of polymerization.

### 3.4 Differences of starch crystalline structure under high natural temperature during the reproductive stage

**Figure 6:**
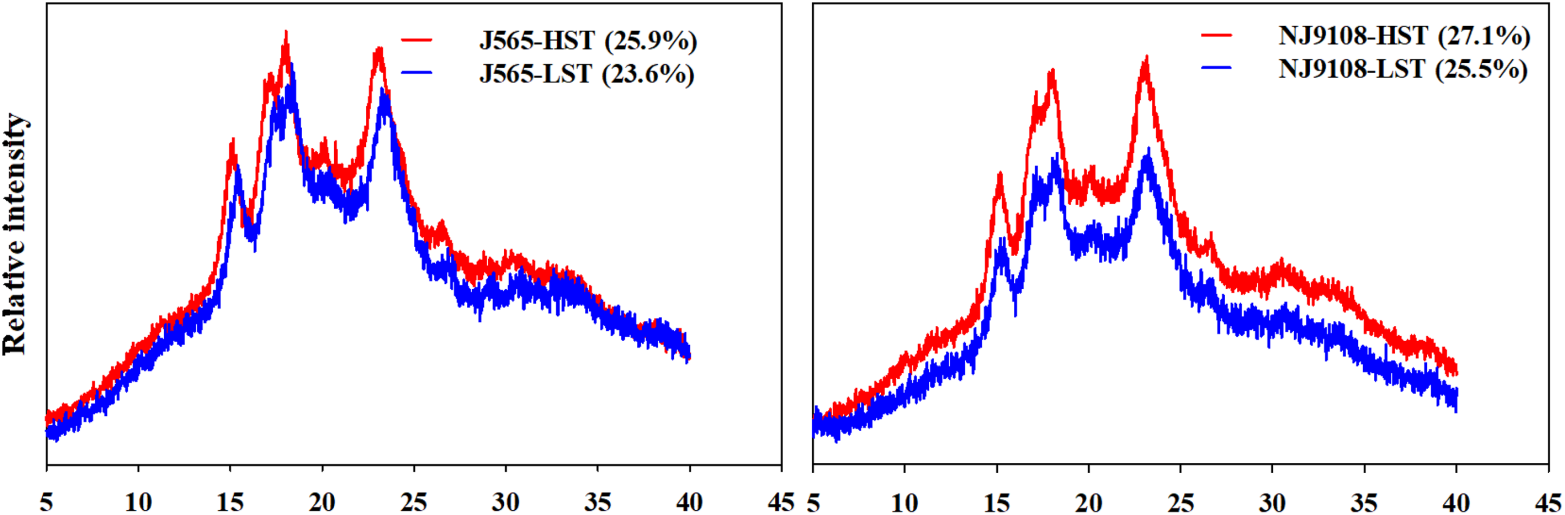
The X-ray diffraction (XRD) and relative crystallinity of starch. The data in brackets are the values of relative crystallinity. HST and LST represent high seasonal temperature and low seasonal temperature, during the reproductive stage in 2017 and 2018. J565 and NJ9108 represent cultivars Jing-565 and Nanjing-9108.

The XRD profiles of rice starch are observed and shown in **Figure 6**. All starch samples presented a typical A-type diffraction pattern. There were two strong peaks at approximately 15° and 23° 2*θ*. Approximately, at 17° and 18° 2*θ*, there was an unresolved doublet. This result indicated that HST did not change the XRD patterns of rice starch. However, the relative crystallinity presented significant differences between HST and LST in the present study. Both cultivars had a greater degree of crystallinity in HST than in LST. These results presented that high natural temperature during the reproductive stage might increase the relative crystallinity of starch.

### 3.5 Relationship of rice quality with starch structure

**Figure 7:**
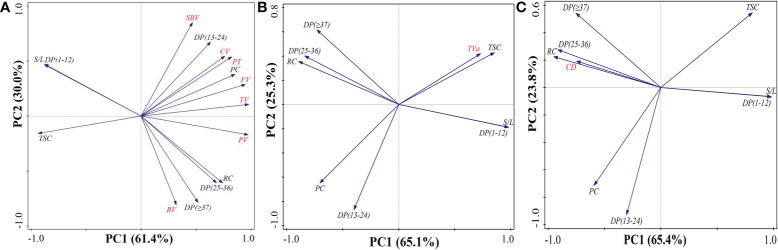
The principal components analysis of total starch content, protein content, and starch structure with rice quality including pasting properties **(A)**, taste value **(B)**, and grain chalkiness degree **(C)**. S/L, rate of short chains (DP < 12) to long chains (DP > 12); TSC, total starch content; PC, protein content; SBV, setback viscosity; TV, trough viscosity; BV, breakdown viscosity; FV, final viscosity; CV, consistence viscosity; PT, pasting temperature; DP, degree of polymerization; RC, relative crystallinity; TVa, taste value; CD, chalkiness degree.

The values of *r* are displayed in **Table 4** to establish the relationship between starch structure, chalkiness, and ECQ. The results showed that chalkiness and ECQ were significantly correlated with chemical composition content (total starch and protein content) and closely associated with starch structure, including relative crystallinity and the fractions of debranched amylopectin. For example, the RVA profiles demonstrated significant negative correlations with total starch content, short chains (DP < 12), and S/L. However, they showed markedly positive correlations with protein content and long chains. These results agree with the finding that taste value was positively correlated with total starch content (*r* = 0.69*, *P < 0.05), short chains (*r* = 0.64*), and S/L (DP > 12) (*r* = 0.65*). However, it was negatively correlated with protein content, relative crystallinity, and long chains. For chalkiness degree, it was negatively correlated with total starch content, short chains, and S/L, but it was positively correlated with protein content, relative crystallinity, and long chains. Furthermore, the principal components analysis demonstrated that PC1 and PC2 explained 91.4%, 90.4%, and 89.2% of the total variations in pasting properties, taste value, and grain chalkiness degree, respectively (**Figure 7**). These results suggested that rice quality was closely correlated not only with chemical composition content but also with starch structure including relative crystallinity and the fractions of debranched amylopectin.

**Table 4:** Correlations of the RVA profile and taste value with chemical composition and starch structure.

	Total starch content	Protein content	Relative crystallinity	DP (1-12)	DP (13-24)	DP (25-36)	DP (≥37)	S/L
Peak viscosity	−0.87**	0.77**	0.81**	−0.93**	0.51	0.75**	0.63*	−0.93**
Trough viscosity	−0.93**	0.85**	0.68*	−0.81**	0.69*	0.62*	0.42	−0.81**
Final viscosity	−0.90**	0.91**	0.51	−0.70*	0.76**	0.45	0.29	−0.70*
Breakdown	−0.12	0.06	0.64*	−0.64*	−0.31	0.59*	0.81**	−0.65*
Setback	−0.53	0.69*	−0.19	−0.43	0.79**	−0.21	−0.36	−0.02
Consistence	−0.72**	0.88**	0.15	−0.42	0.77**	0.10	0.05	−0.42
Pasting temperature	−0.87**	0.92**	0.28	−0.47	0.85**	0.26	0.01	−0.47
Taste value	0.69*	−0.83**	−0.39	0.64*	−0.54	−0.30	−0.42	0.65*
Chalkiness degree	−0.53	0.78**	0.21	−0.64*	0.08	0.82**	0.46	−0.63*

DP, degree of polymerization; S/L, rate of short chains (DP < 12) to long chains (DP > 12).

* and ** indicate significance at the P < 0.05 and P < 0.01 levels, respectively (n = 12).

## 4 Discussion

### 4.1 Response of rice quality to high natural temperature during the reproductive stage

Chalkiness and ECQ directly affect the economic value of rice and are significantly influenced by environmental stress (Deng et al., 2018; Chen et al., 2021). In the present study, compared with LST, HST significantly increased the chalkiness degree (**Figure 2**). Similarly, previous findings demonstrated that high temperature during the reproductive stage would increase rice chalkiness (Cheabu et al., 2018; Zhen et al., 2019). In addition, the RVA profiles of rice flour under two contrasting natural temperature conditions were quite different (**Figure 3**). Compared with HST, the rice flour of LST demonstrated lower pasting temperature, setback, and consistence, which were closely associated with high ECQ (Yan et al., 2016; Zhu et al., 2021a). The taste meter is equipped with a prediction model based on the characteristics of Chinese rice. Therefore, the taste values were valuable to evaluate the quality of rice (Zhang et al., 2016a; Chen et al., 2021), and the results showed that HST significantly declined the taste value (**Figure 2**). These findings suggested that high natural temperature during the reproductive stage significantly increased the rice chalkiness as well as reduced the ECQ.

There is no doubt that solar radiation significantly affects crop production and quality; for example, shading stress significantly decreased maize quality (Shi et al., 2018) and greatly increased rice chalkiness degree (Deng et al., 2018). However, we found that both cultivars under high solar radiation during the reproductive stage had a significantly higher chalkiness and lower taste value (**Supplementary Table S1**; **Figure 2**). This might be contributed by the fact that high temperature is often accompanied by higher solar radiation, and the former during the reproductive stage markedly degraded rice quality (Cheabu et al., 2018; Zhen et al., 2019; Lin et al., 2020). In addition, numerous studies suggested that temperature is the most important climatic factor governing rice growth (Huang et al., 2013; Deng et al., 2015). Hence, the differences in chalkiness and ECQ were mainly caused by temperature changes during the reproductive stage in this study.

### 4.2 Chemical composition content and starch structure in response to high natural temperature during the reproductive stage

Concurring with our observations, previous research showed that high temperature during the reproductive stage significantly decreased the apparent amylose content (Lin et al., 2020). However, the AAC showed slight differences between HST and LST in the present study (**Figure 4**). Two possible explanations for this difference were observed: 1) previous studies were conducted under controlled temperature chambers, but it does not necessarily reproduce field conditions (Huang et al., 2013); and 2) the effects of high temperature on the AAC are cultivar-dependent (Cheabu et al., 2018; Yao et al., 2019). However, the total starch and protein content were significantly different between HST and LST. HST significantly increased the protein content. It was consistent with the previous finding that high temperature before heading greatly elevated grain protein content (Cheabu et al., 2018; Zhen et al., 2019). These results were mainly induced by the reduction of assimilate supply (**Supplementary Figure S2**). The reason for this result might be induced by the low LAI and low specific leaf weight (**Supplementary Table S2**). This reduction would induce poor sink activity, i.e., poor activities of several starch synthase enzymes (Li et al., 2017), and finally inhibit endosperm cell development and starch deposition (Fu et al., 2011; Lin et al., 2020). Therefore, the above results revealed that high natural temperature during the reproductive stage would induce the inconsistent development of starch granules and protein bodies by the changes in the source. As a result, these eventually might result in degrading rice quality such as increasing chalkiness and low taste value (Zhang et al., 2016a; Tang et al., 2019; Tu et al., 2022).

Furthermore, the starch structure also demonstrated significant differences between the two growth seasons in the present study (**Figures 5**, **6**). For example, the proportion of short chains was significantly lower in HST than in LST, but the relative amount of intermediate chains (B1 and B2) was higher in HST. The reason for this result might be that the activities of soluble starch synthase (SSS) isoforms were upregulated by abiotic stress such as high-temperature stress (Liao et al., 2015; Timabud et al., 2016). Several studies have proven that they play important roles in the synthesis of external segments of B chains (McMaugh et al., 2014; Chen and Bao, 2016). For crystallinity, all starch presented a typical A-type diffraction pattern, which is consistent with the XRD profiles of cereal starch (Zhu et al., 2021b). This result indicated that HST did not change the XRD patterns of rice starch. Similarly, numerous studies demonstrated that there were no variations of the XRD patterns in response to environments and management, such as shading (Shi et al., 2018), temperature (Lin et al., 2020), and nitrogen fertilizer (Tang et al., 2019). Instead, the relative crystallinity presented significant differences between HST and LST in the present study. It is generally considered that amylopectin determined the starch crystallinity, while amylose influenced the crystalline packing of amylopectin (Cai et al., 2015; Zhu et al., 2021b). However, these results showed slighter differences in amylose content between HST and LST (**Figure 4B**). Therefore, in the present study, the starch samples had high relative crystallinity mainly due to increased numbers of B1 and B2 chains under high natural temperature during the reproductive stage. It was in agreement with previous reports that the increase in the intermediate amylopectin chain induced increased crystallinity (Zhang et al., 2016a; Shi et al., 2018). These results suggested that not only chemical composition content but also starch structure demonstrated significant changes under high natural temperature during the reproductive stage.

### 4.3 Relationships of rice quality with chemical composition content and starch structure

Numerous studies indicated that not only amylose and protein content but also the fine structure of amylopectin determined the formation of rice quality (Cai et al., 2015; Yao et al., 2019; Shi et al., 2022). It was consistent with our finding that starch structure (chain length distribution and crystallinity), total starch content, and protein content explained 91.4%, 90.4%, and 89.2% of the total variations in pasting properties, taste value, and grain chalkiness degree, respectively (**Figure 7**). The starch structure may affect the interaction between starch chains within the amorphous and crystalline domains (Cai et al., 2015; Zhang et al., 2016a). This interaction might result in differences in the swelling power, solubility, and retrogradation of rice grain (Zhu et al., 2016; Chen et al., 2021) and eventually induce changes in pasting viscosity. In the present study, there were slight changes in amylose content but great variations in protein content, distribution of amylopectin chain length, and degree of crystallinity. Thus, the increased pasting viscosity of rice starch under HST was primarily ascribed to high protein content (Chen et al., 2021), high crystallinity, and low short-chain (DP < 12) to long-chain (DP > 12) rate (Zhang et al., 2016a; Shi et al., 2018). Increased long amylopectin chains might contribute to the high pasting temperature in HST. Previous research has proven that starch with long chains requires higher temperatures for complete dissociation (Zhang et al., 2016a; Zhu et al., 2016). Therefore, these findings suggested that variations in rice quality were closely associated not only with changes in chemical composition content but also with starch structure in response to high natural temperature during the reproductive stage.

## 5 Conclusion

It is necessary to clearly understand the influences of high natural temperature on the structural and physicochemical properties of rice starch. Our results demonstrated that HST significantly increased grain chalkiness, setback, consistence, and pasting temperature and reduced rice taste values. The variations in rice quality were closely associated not only with the reduced total starch and increased protein content but also with the altered starch structure including reduced short chains (DP < 12), increased long chains (DP > 12), and relative crystallinity. These results suggested that changes in starch structure also played an important role in determining rice quality under high natural temperature during the reproductive stage. These findings highlighted the importance of considering the effect of high natural temperature during the reproductive stage on the structure and physicochemical properties of rice starch in further breeding and practice.

## Data availability statement

The original contributions presented in the study are included in the article/**Supplementary Material**. Further inquiries can be directed to the corresponding authors.

## Author contributions

CC, WW, and DT initiated and designed the research, analyzed the data, and wrote the manuscript. DT, XS, and BC performed the experiments. YJ, AS, WW, MX and MC revised and edited the manuscript and also provided advice on the experiments. All authors contributed to the article and approved the submitted version.
