# Supplementary material for: Variation of rice starch structure and physicochemical properties in response to high natural temperature during the reproductive stage
Source: Front Plant Sci. 2023 Feb 14;14:1136347. doi: 10.3389/fpls.2023.1136347 (PMC9971927; doi:10.3389/fpls.2023.1136347)
Supplement: Supplementary file 1 [file DataSheet_1.docx]

Supplementary Material

Variation of rice starch structure and physicochemical properties in response to high natural temperature during reproductive stage

**Debao Tu^1,2^, Yang** **Jiang^2^,** **Akram Salah^2^, Min Xi^1^, Mingli** **Cai^2^, Bo Cheng^2^, Xiaosong Sun^3^, Cougui Cao^2,^*, Wenge Wu ^1,^***

*** Correspondence: Cougui Cao ccgui@mail.hzau.edu.cn;**

**Wenge Wu wuwenge@vip.sina.com**

**1 Supplementary Data**

**Supplementary Figure S1** Mean Daily temperature conditions for rice growth season in 2017 and 2018.

**Supplementary Figure S2** Differences of matter translocation of stem-sheath in different treatment between two contrasting natural field temperature conditions.

**Supplementary Table S1** Mean solar radiation (MJ m^−2^d^−1^) during different phenological stages in different situations

**Supplementary Table S2** Difference of characteristic of rice source and sink between two contrasting natural field temperatures conditions.

**2 Supplementary Figures and Tables**

**2.1 Supplementary Figures**


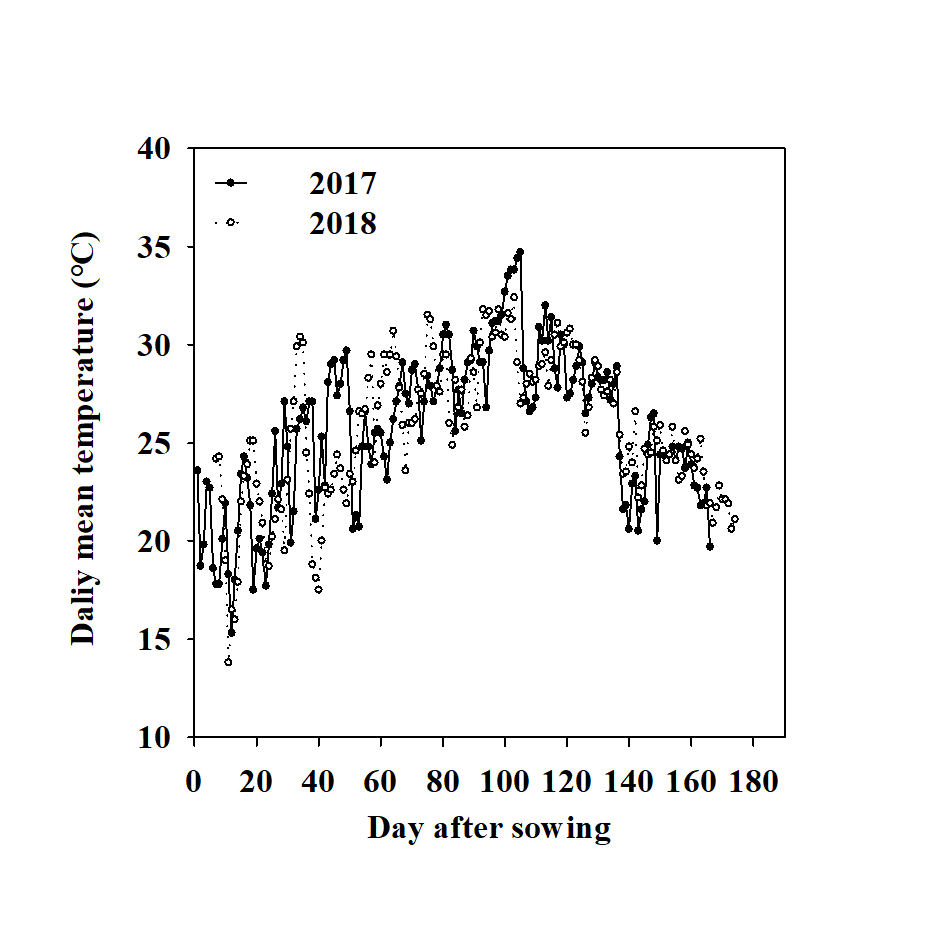


**Fig. S1.** Mean Daily temperature conditions for rice growth season (A) and during reproductive stage (B and C) in 2017 and 2018.


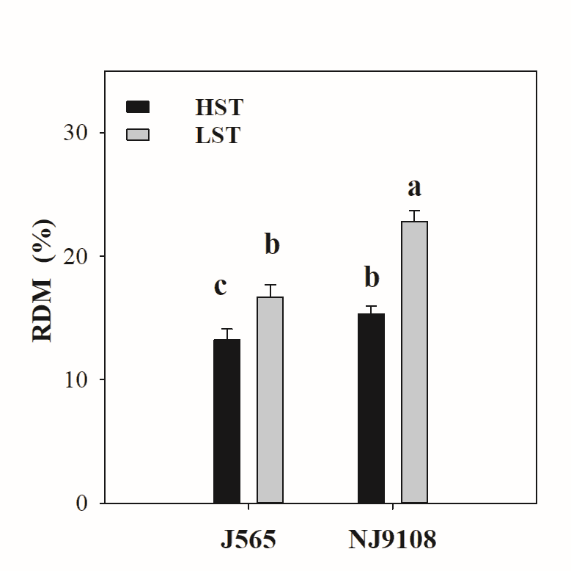


**Fig. S2** Differences of matter translocation of stem-sheath in different treatment between two contrasting natural field temperature conditions. RDM: Ration of dry matter translocation to grain. Different letters present significant difference according to the LSD (0.05). HST and LST represent high seasonal temperature and low seasonal temperature, during reproductive stage in 2017 and 2018. J565 and NJ9108 represent cultivars Jing 565 and Nanjing 9108, respectively. Different letters indicate significant difference at P＜0.05.

**2.2 Supplementary Tables**

| **Table S1 Mean solar radiation (MJ m^−2^d^−1^) during different phenological stages in different situations.** | | | |
| --- | --- | --- | --- |
| Treatme | VR | RR | GR |
| J565-HST | 15.7 | 18.1 | 14.9 |
| J565-LST | 15.7 | 17.9 | 14.9 |
| NJ9108-HST | 15.6 | 17.7 | 14.9 |
| NJ9108-LST | 15.7 | 17.6 | 15.0 |
| HST and LST represent high seasonal temperature and low seasonal temperature, during reproductive stage in 2017 and 2018. VR, RR and GR represent mean solar radiation during vegetative stage, reproductive stage and grain filling stage, respectively. J565 and NJ9108 represent cultivars Jing 565 and Nanjing 9108, respectively. | | | |

| **Table S2 Difference of characteristic of rice source and sink between two contrasting natural field temperatures conditions.** | | | | |
| --- | --- | --- | --- | --- |
| Treatment | LAI  (m^2^m^-2^) | SLW  (mg cm^-2^) | Leaf /sink ratio  (cm^2^ g^-1^) | Stem-sheath /sink ratio  (mg g^-1^) |
| J565-HST | 6.23 ± 0.05 b | 5.03 ± 0.09 b | 49.8 ± 1.49 a | 530.3 ± 19.07 a |
| J565-LST | 6.77 ± 0.25 a | 6.47 ± 0.15 a | 42.2 ± 2.65 b | 511.1 ± 13.77 ab |
| NJ9108-HST | 6.32 ± 0.05 b | 5.03 ± 0.19 b | 42.4 ± 0.19 b | 473.9 ± 33.86 b |
| NJ9108-LSRT | 7.43 ± 0.81 a | 6.30 ± 0.09 a | 44.5 ± 2.39 b | 549.1 ± 17.16 a |
| HST and LST represent high seasonal temperature and low seasonal temperature, during reproductive stage in 2017 and 2018. LAI：Leaf area index；SLW：Specific leaf weight. Different letters denote significant difference within the same column according to the LSD (0.05). J565 and NJ9108 represent cultivars Jing 565 and Nanjing 9108, respectively. | | | | |
